# Supplementary material for: Disruption of the SAGA CORE triggers collateral degradation of KAT2A
Source: Nat Commun. 2026 Apr 20;17:3410. doi: 10.1038/s41467-026-71613-7 (PMC13096341; doi:10.1038/s41467-026-71613-7)
Supplement: Supplementary file 1 — Supplementary Information [file 41467_2026_71613_MOESM1_ESM.pdf]

Supplementary Information

Disruption of the SAGA CORE triggers collateral degradation of KAT2A

Paul Batty[1,2], Hannah Beneder[1,2], Caroline Schätz[2], Gabriel Onea[2,3], Maciej Zaczek[1,2], Ana P. Kutschat[1,2], Miriam Abele[2], Sophie Müller[1,2], Giulio Superti-Furga[2,4], Georg E. Winter[2,5], Davide Seruggia[1,2 *]

[1]St. Anna Children's Cancer Research Institute (CCRI), 1090 Vienna, Austria

[2]CeMM Research Center for Molecular Medicine of the Austrian Academy of Sciences, 1090 Vienna, Austria

[3]Department of Pediatrics and Adolescent Medicine, Medical University of Vienna, 1090 Vienna, Austria

[4]Center for Physiology and Pharmacology, Medical University of Vienna, 1090 Vienna, Austria

[5]AITHYRA Institute for Biomedical Artificial Intelligence, 1030 Vienna, Austria

*Correspondence: davide.seruggia@ccri.at

Inventory of Supplementary Information

• Supplementary Figures and Supplementary Figure Legends

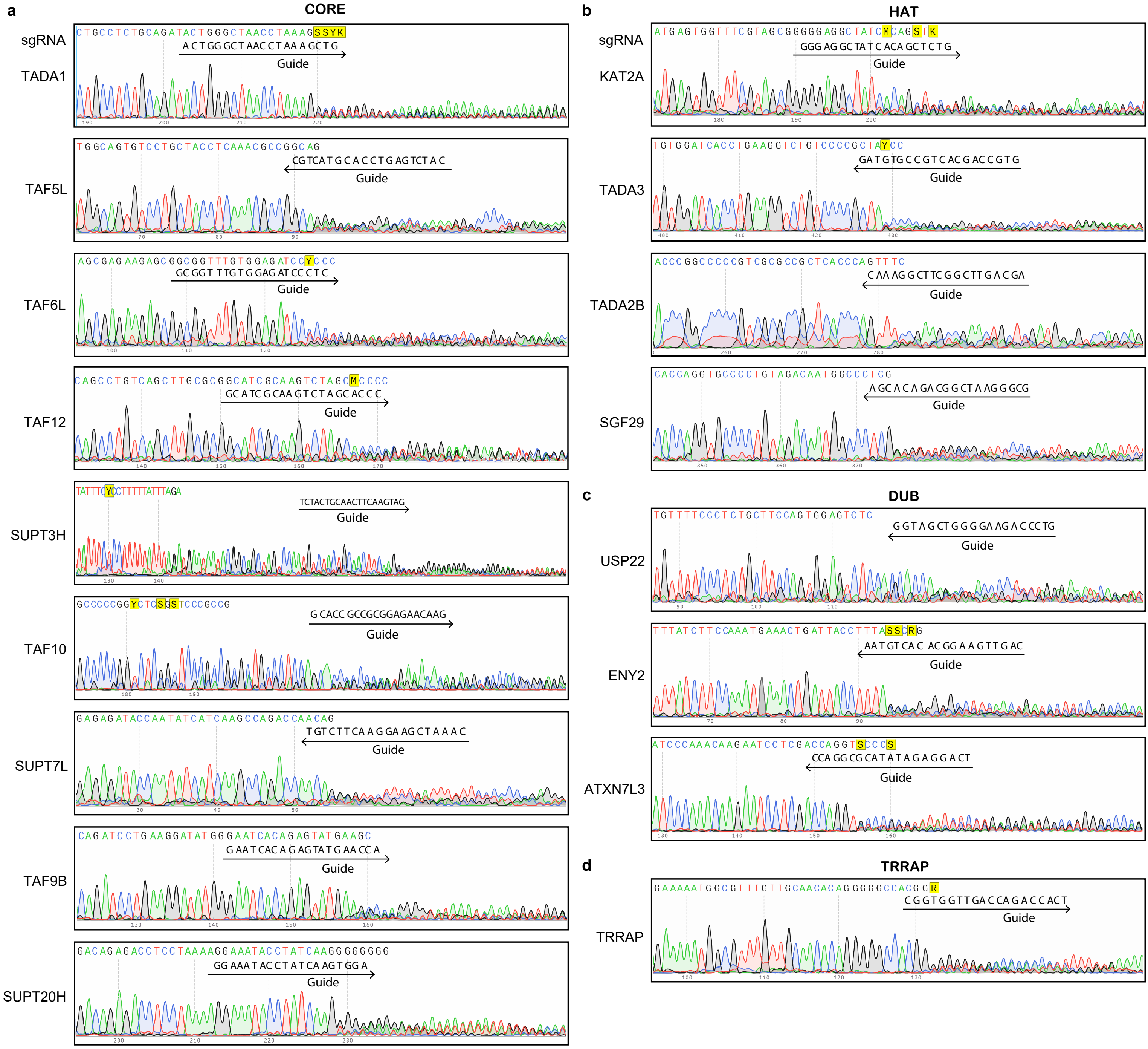

Supplementary Figure 1 | Validation of editing efficiency of SAGA sgRNA library

a-d) Sanger sequencing tracks of loci around sgRNA target sites show strong and comparable editing. Cas9 expressing cells were transduced with eGFP-expressing sgRNAs targeting the **a)** CORE, **b)** HAT, **c)** DUB and **d)** TRRAP modules of SAGA. eGFP positive cells were sorted by FACS, gDNA extracted and the loci around the editing sites amplified by PCR before Sanger sequencing. The position of the sgRNA binding site is annotated on the sequencing track for each locus.

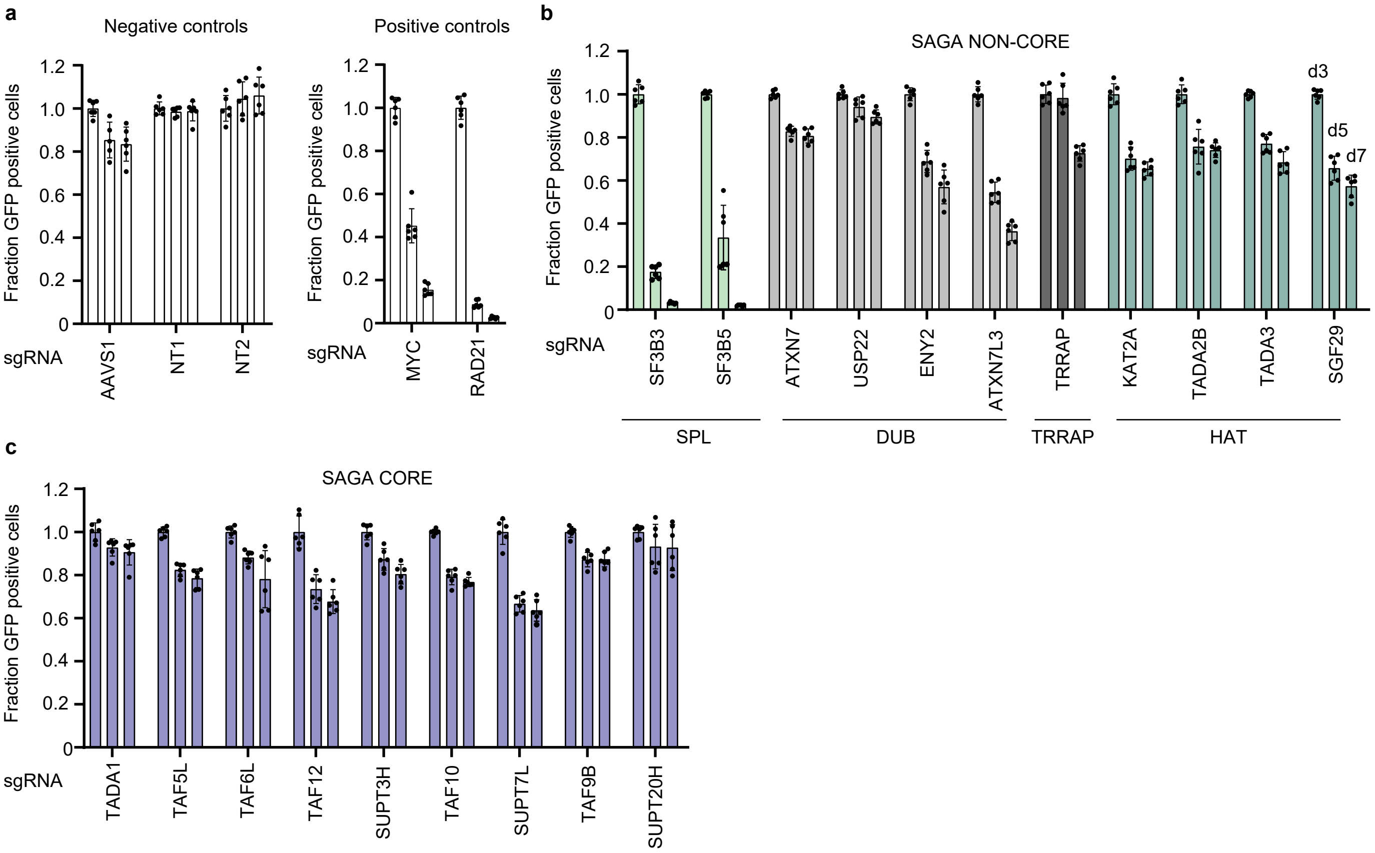

Supplementary Figure 2 | Guide RNA dropout of SAGA sgRNA library

a-c) Assessment of sgRNA dropout for each guide in the arrayed SAGA-focused sgRNA library. The fraction of eGFP positive cells for each guide was measured 3, 5, and 7 days post-transduction of wild type HAP1^{Cas9} cells expressing the KAT2A stability reporter. The fraction of eGFP positive cells was normalised to the day 3 value for each guide. SAGA CORE components are visualised in purple, histone acetyltransferase (HAT) components in dark green, splicing components (SPL) in light green, deubiquitinase (DUB) components in light grey, TRRAP in dark grey, positive and negative controls in white. Negative controls: AAVS1, NT1 (non-targeting 1), NT2 (non-targeting 2). Day 3 (d3), day 5 (d5), day 7 (d7) data are plotted from left to right for each guide. Black dots represent the fraction of eGFP positive cells for biological replicates on each day, error bars indicate standard deviation, bars indicate the mean for each condition. **a)** Fraction of eGFP positive cells for positive and negative control sgRNAs. **b)** Fraction of eGFP positive cells for SAGA non-CORE module sgRNAs. **c)** Fraction of eGFP positive cells for SAGA CORE module sgRNAs. Biological replicates: a-c (n = 6).

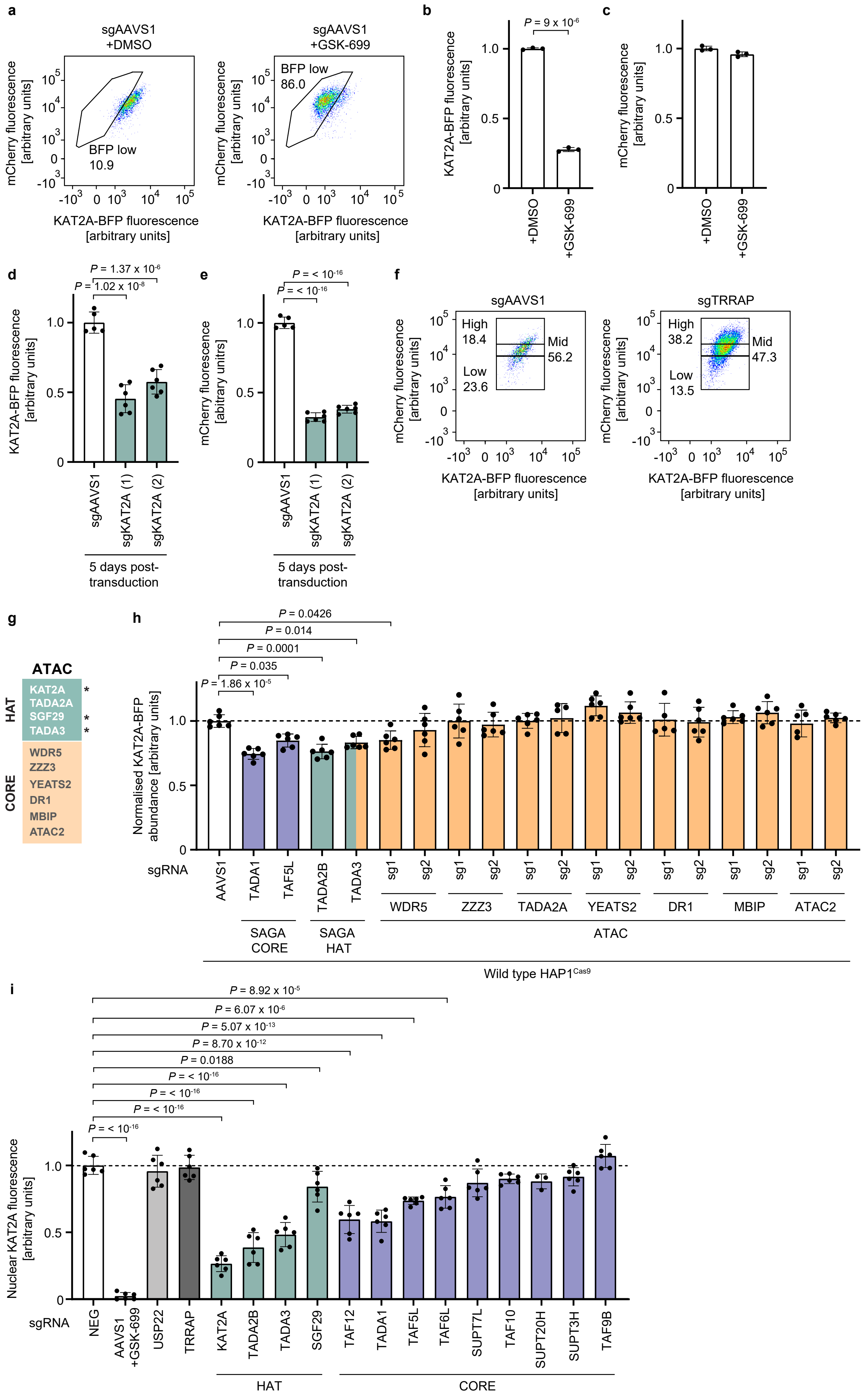

Supplementary Figure 3 | Responsiveness of the KAT2A stability reporter to chemical and genetic perturbation

Legend on next page.

Supplementary Figure 3 | Responsiveness of the KAT2A stability reporter to chemical and genetic perturbation

a) Scatter plot of KAT2A-BFP vs mCherry fluorescence for wild type HAP1^{Cas9} cells expressing the KAT2A stability reporter after treatment with DMSO or GSK-699. **b, c)** Quantification of KAT2A-BFP **(b)** or mCherry **(c)** fluorescence by flow cytometry for cells as in **a** following treatment with DMSO or GSK-699. **d, e)** Quantification of KAT2A-BFP **(d)** or mCherry **(e)** fluorescence by flow cytometry for cells as in **a**, 5 days post-transduction with sgRNAs targeting *AAVS1* (white bars) or *KAT2A* (green bars). eGFP positive cells were analysed. **f)** Scatter plot of KAT2A-BFP vs mCherry fluorescence for cells as in **a**, 5 days post-transduction with sgRNAs against *AAVS1* or *TRRAP*. Black boxes mark mCherry low, mid, and high populations. **g)** Schematic of ATAC complex subunits. Shared SAGA/ATAC HAT components are indicated with an asterix. **h)** Quantification of KAT2A-BFP protein abundance by flow cytometry 5 days post-transduction of sgRNAs targeting ATAC subunits in wild type HAP1^{Cas9} cells expressing the KAT2A stability reporter. ATAC (orange), SAGA HAT (green) and SAGA CORE (purple) components are visualised. TADA3 is common to ATAC and SAGA and is visualised in both colours. **i)** Quantification of nuclear KAT2A fluorescence by immunofluorescence following transduction with the indicated sgRNAs, as in Fig. 1e. SAGA CORE components (purple), HAT components (green), USP22 (light grey), TRRAP (dark grey), and negative controls (NEG, AAVS1 + GSK-699) (white) are visualised. a, d-f, h, i) eGFP positive cells were analysed. Biological replicates: d, e, h, i (n = 6); b, c (n = 3). Technical replicates: b, c (n =3). b, d, h, i) Dots represent the mean of each biological replicate, error bars indicate standard deviation; bars indicate the mean for each condition. b) Significance was tested using a two-tailed, unpaired Welch's t-test. d, e, h, i) Significance was tested using a one-way ANOVA with a post-hoc Dunnett's multiple comparison test. Exact p-values are specified in the figure.

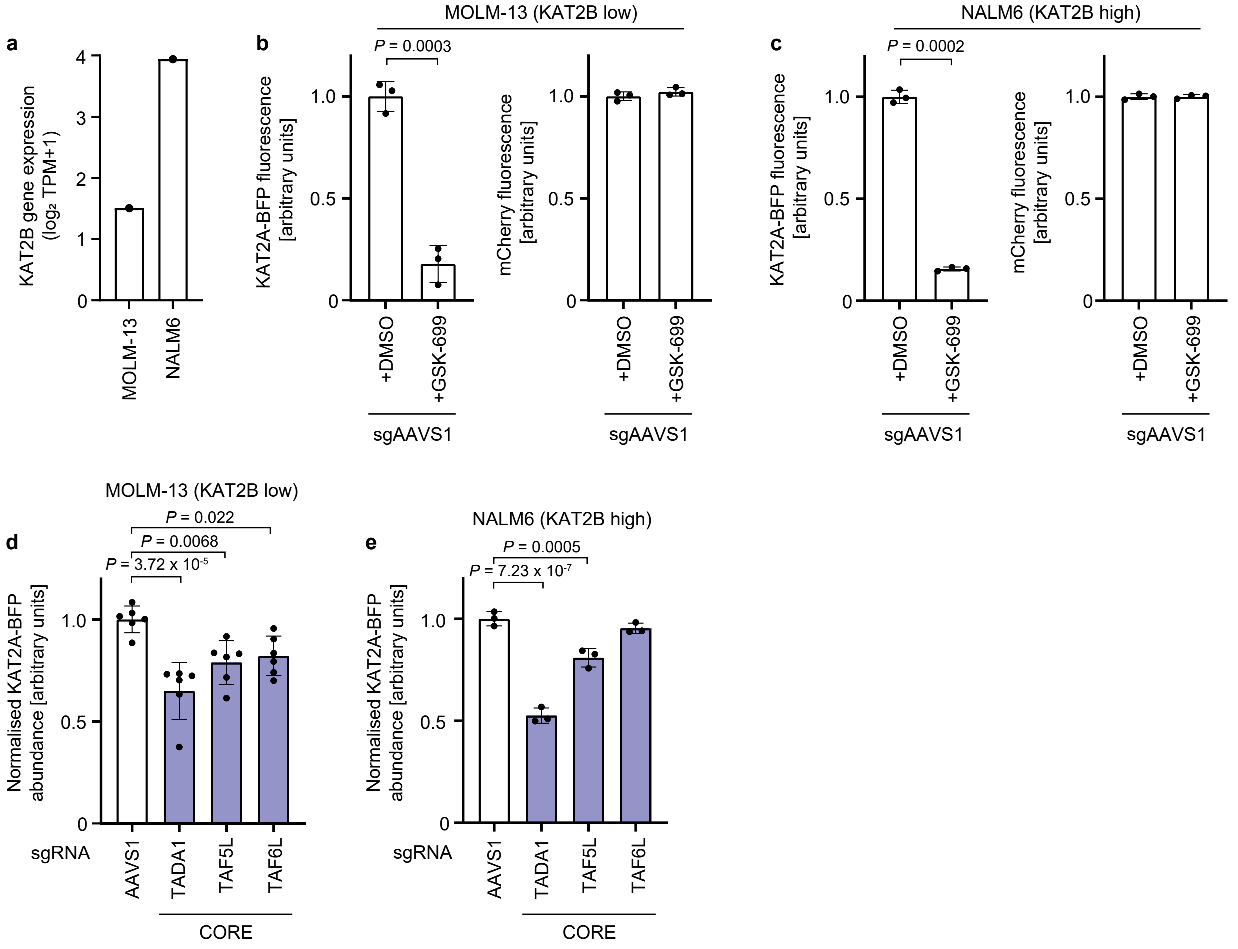

Supplementary Figure 4 | Perturbation of the SAGA CORE reduces KAT2A protein abundance in leukaemia subtypes

a) DepMap gene expression data of KAT2B (public dataset 25Q3) for KAT2B-low (MOLM-13) and KAT2B-high (NALM6) expressing leukaemia cell lines. **b, c)** Validation of the responsiveness of MOLM-13^{Cas9} (**b**) or NALM6^{Cas9} (**c**) cells expressing the KAT2A stability reporter to chemical perturbation. Cells were transduced with eGFP-expressing sgRNAs targeting *AAVS1*. 5 days post-transduction cells were treated with DMSO or GSK-699 before analysis by flow cytometry. eGFP positive cells are plotted. **d, e)** Perturbation of the SAGA CORE reduces KAT2A protein abundance in leukaemia cells. MOLM-13^{Cas9} (**d**) or NALM6^{Cas9} (**e**) cells expressing the KAT2A stability reporter were transduced with eGFP-expressing sgRNAs against SAGA CORE components (*TADA1, TAF5L, TAF6L*, purple bars) or *AAVS1* (white bar). 5 days (MOLM-13) or 9 days (NALM6) post-transduction, the KAT2A-BFP levels of eGFP positive cells were quantified using flow cytometry. Biological replicates: b, c, e (n = 3); d (n = 6). a) Dots represent mean KAT2B expression. b-e) Dots represent the mean of biological replicates. Error bars indicate standard deviation; bars indicate the mean for each condition. b, c) Significance was tested using a two-tailed, unpaired Welch's t-test. d, e) Significance was tested using a one-way ANOVA with a post-hoc Dunnett's multiple comparison test. Exact p-values are specified in the figure.

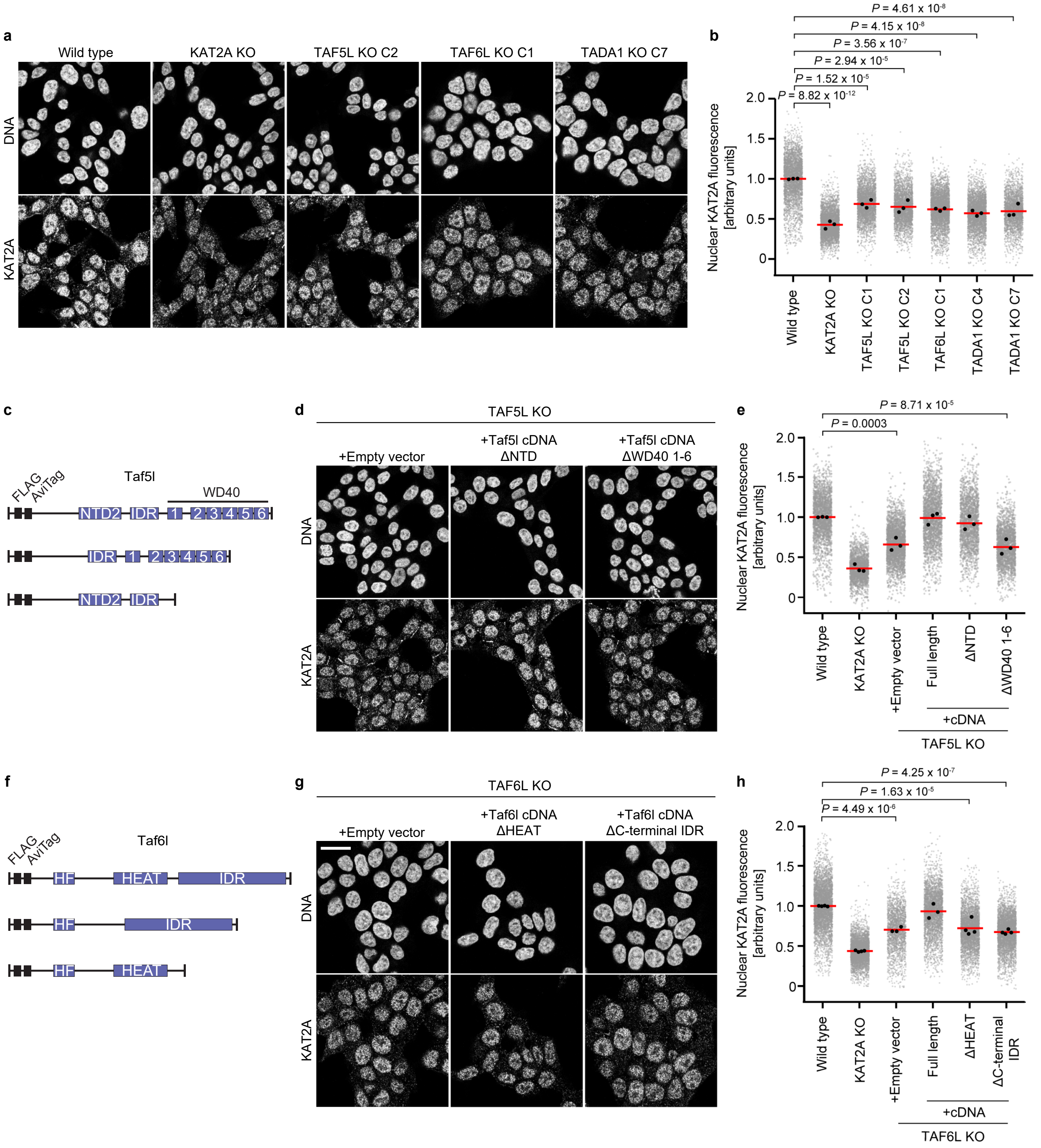

Supplementary Figure 5 | Specific domains of the SAGA CORE regulate KAT2A protein abundance

a) Representative immunofluorescence images of KAT2A and DNA for wild type or KO HAP1 cells as indicated. **b)** Quantification of nuclear KAT2A fluorescence by immunofluorescence for conditions as indicated. **c)** Schematic of murine TAF5L cDNA overexpression constructs. **d)** Representative immunofluorescence images of KAT2A and DNA for TAF5L KO cells overexpressing Taf5l cDNA overexpression constructs as indicated. **e)** Quantification of nuclear KAT2A fluorescence by immunofluorescence for conditions as indicated. **f)** Schematic of murine TAF6L cDNA overexpression constructs. **g)** Representative immunofluorescence images of KAT2A and DNA for TAF6L KO cells overexpressing Taf6l cDNA overexpression constructs as indicated. **h)** Quantification of nuclear KAT2A fluorescence by immunofluorescence for conditions as indicated. Biological replicates: a, b, d, e, (n = 3); g, h (all conditions n = 4, apart from TAF6L KO$^{\text{empty vector}}$, TAF6L KO$^{\text{+cDNA full length}}$ (n = 3)). b, e, h) Grey dots represent the mean of individual nuclei, black dots indicate the mean of each biological replicate, red bars indicate the mean of biological replicates. Significance was tested using a one-way ANOVA with a post-hoc Dunnett's multiple comparison test. Exact p-values are specified in the figure. All images show single Z-slices. To aid visualisation the DNA channel is not contrast matched. Scale bars: 20 µm.

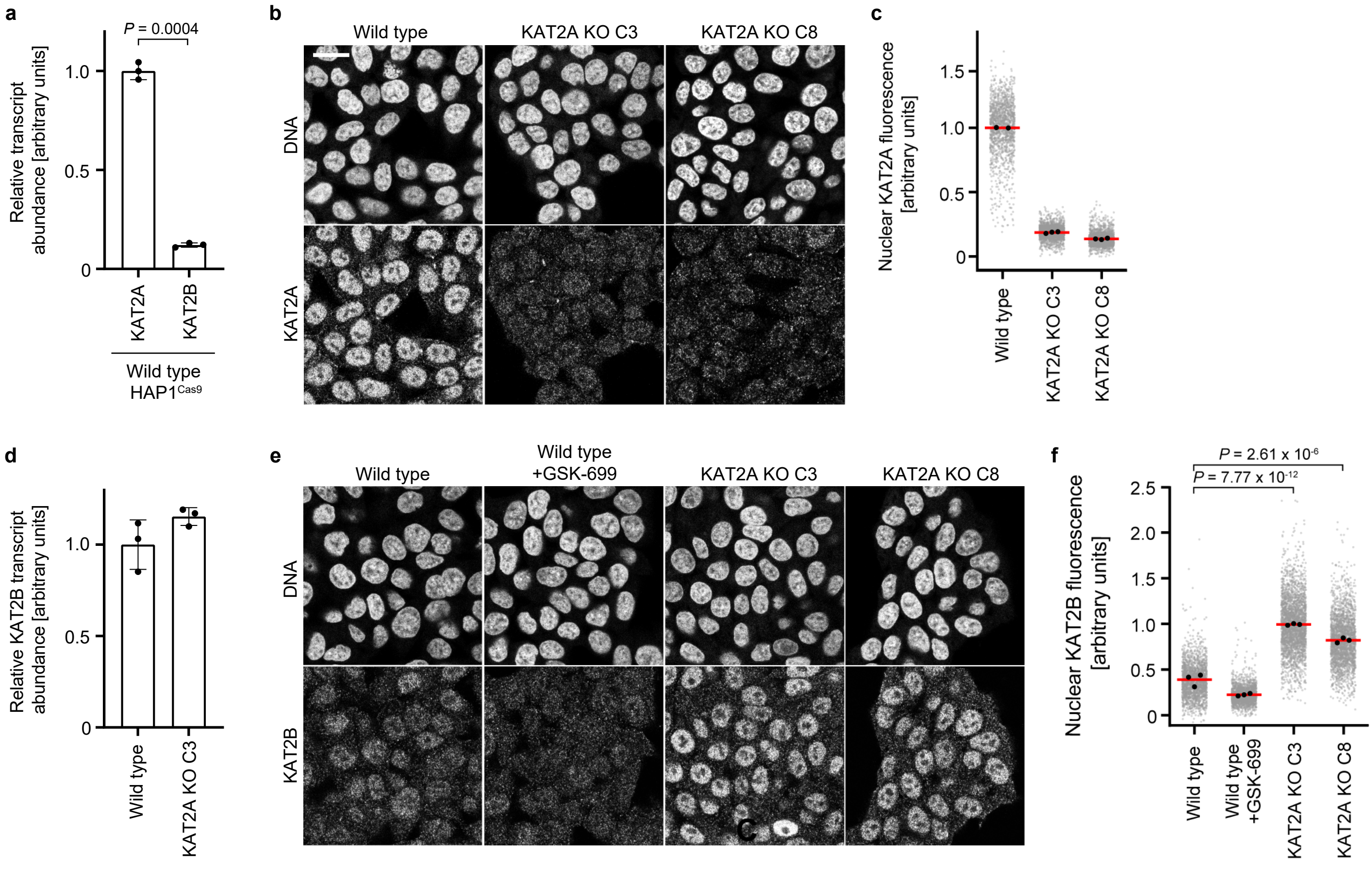

Supplementary Figure 6 | KAT2B is lowly expressed in HAP1 cells and upregulated in the absence of KAT2A

a) RT-qPCR of KAT2A and KAT2B mRNA transcript levels in wild type HAP1 cells. **b)** Representative immunofluorescence images of KAT2A and DNA for wild type or KAT2A KO cells as indicated. **c)** Quantification of nuclear KAT2A fluorescence by immunofluorescence for conditions as indicated. **d)** RT-qPCR of KAT2B mRNA transcript levels in wild type and KAT2A KO HAP1 cells. **e)** Representative immunofluorescence images of KAT2B and DNA for wild type or KAT2A KO cells as indicated. **f)** Quantification of nuclear KAT2B fluorescence by immunofluorescence for conditions as indicated. Biological replicates: a-f (n = 3, apart from b, c (wild type (n = 2)). Technical replicates: a, d (n =3). c, f) Grey dots represent the mean of individual nuclei, red bars indicate the mean of biological replicates. a, c, d, f) Black dots indicate the mean of each biological replicate. a, d) Error bars represent standard deviation, bars indicate the mean for each condition. a) Significance was tested using a two-tailed, unpaired Welch's t-test. f) Significance was tested using a one-way ANOVA with a post-hoc Dunnett's multiple comparison test. Exact p-values are specified in the figure. All images show single Z-slices. To aid visualisation the DNA channel is not contrast matched. Scale bars: 20 µm.

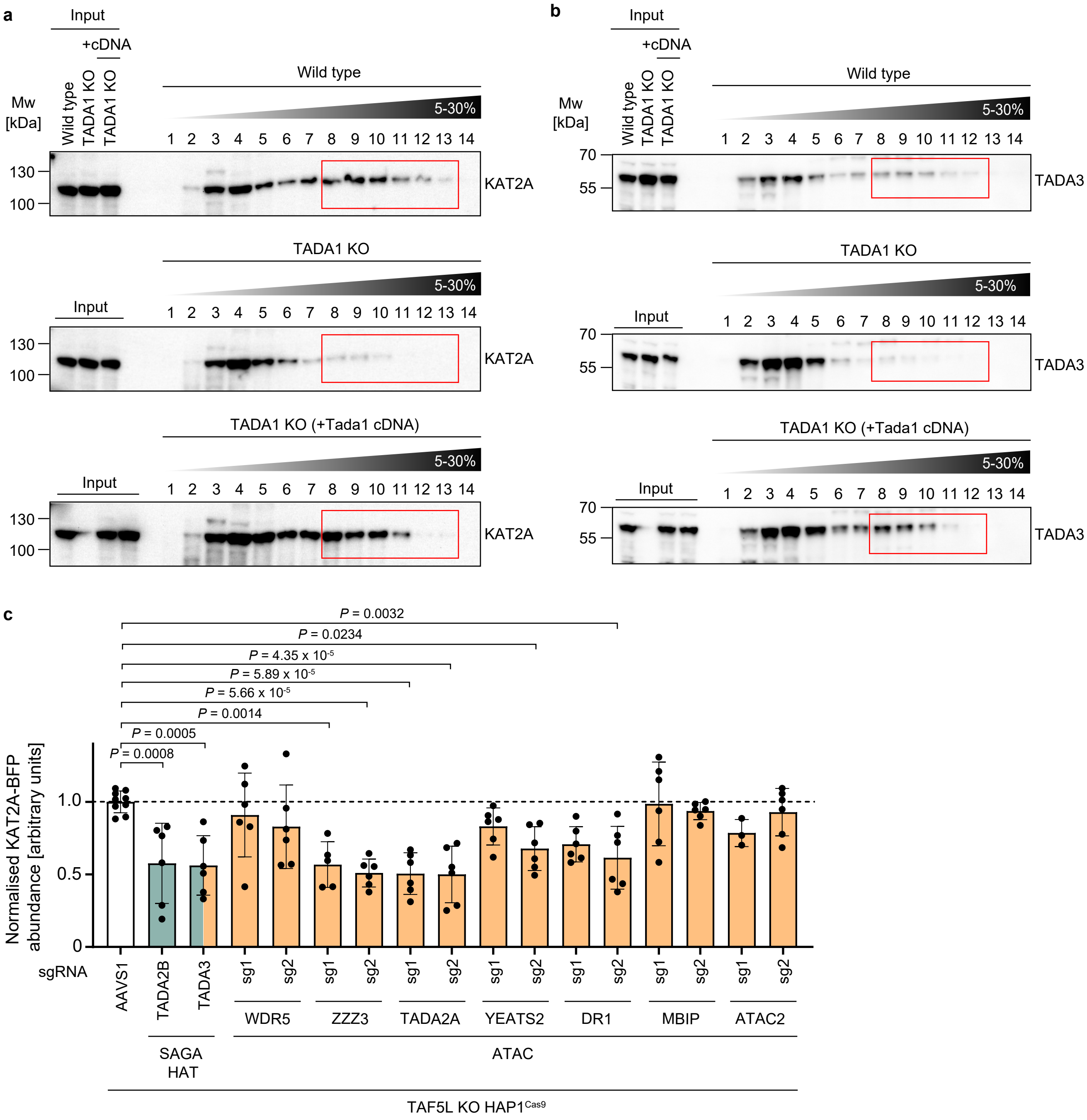

Supplementary Figure 7 | Overexpression of Tada1 cDNA restores KAT2A and TADA3 to high molecular weight fractions

a, b) Cell lysates were prepared from wild type, TADA1 KO, and TADA1 KO cells stably overexpressing Tada1 cDNA, and loaded onto 5 – 30 % sucrose gradients. Gradients were ultracentrifuged and fractionated to separate protein complexes based on their sedimentation. The resultant fractions were loaded onto an SDS-PAGE gel and analysed by western blotting, using antibodies against KAT2A **(a)**, or TADA3 (**b**) as indicated. Membranes were developed simultaneously to allow cross-comparison between genotypes. Inputs indicate the cell lysates for each condition prior to ultracentrifugation. Numbers indicate each fraction, moving from lowest to highest density sucrose fractions (5 – 30 %). Red boxes indicate fractions containing high-molecular weight KAT2A or TADA3 species. **a)** Immunoblot analysis of KAT2A following sucrose gradient fractionation for conditions as indicated. **b)** Immunoblot analysis of TADA3 following sucrose gradient fractionation for conditions as indicated. **c)** Quantification of KAT2A-BFP protein abundance by flow cytometry following transduction of sgRNAs targeting ATAC subunits in TAF5K KO HAP1^{Cas9} cells expressing the KAT2A stability reporter. eGFP positive cells were analysed 5 days post-transduction. Black dots represent the mean of biological replicates, error bars represent standard deviation, bars indicate the mean for each condition. ATAC (orange) and SAGA HAT (green) components are visualised. TADA3 is common to ATAC and SAGA and is visualised in both colours. Biological replicates: a, b (n = 2); c (n = 6). Significance was tested using a one-way ANOVA with a post-hoc Dunnett's multiple comparison test. Exact p-values are specified in the figure.

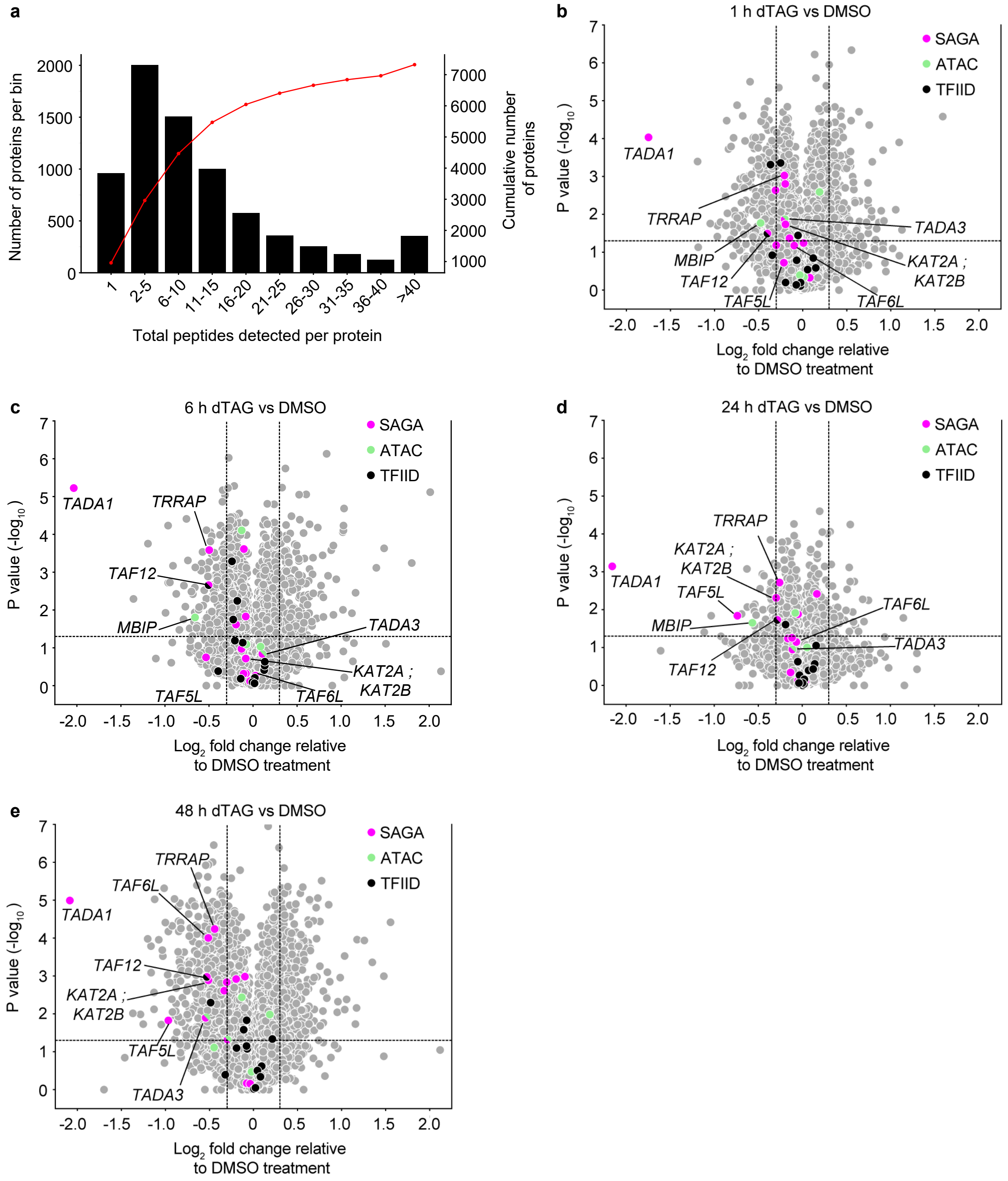

Supplementary Figure 8 | Differentially abundant proteins following acute depletion of TADA1

a) Binned peptide counts for each protein detected in the TMT-expression dataset, plotted as a histogram. The number of proteins within each bin is plotted on the left Y-axis. The cumulative number of identified proteins is plotted on the right Y-axis (red line). **b – e)** Volcano plots of differentially abundant proteins following acute depletion of TADA1 for the indicated time points (1, 6, 24, or 48 h dTAG^{v}-1 treatment) in TADA1-dTAG HAP1 cells. Fold changes and p-values were calculated by comparison of dTAG^{v}-1 treated cells for each time point with DMSO-treated controls. Significantly downregulated proteins: Log_2 fold change ≤ 0.3 and $-log_{10}$ p-values ≥ 1.3. SAGA components (magenta dots), ATAC components (green dots) and TFIID components (black dots) are highlighted. Subunits common to more than one complex are labelled with both colours. Components of the SAGA complex with significantly reduced abundance after 24 h and/or 48 h dTAG treatment are labelled with black lines. Biological replicates: a-e (n = 3).

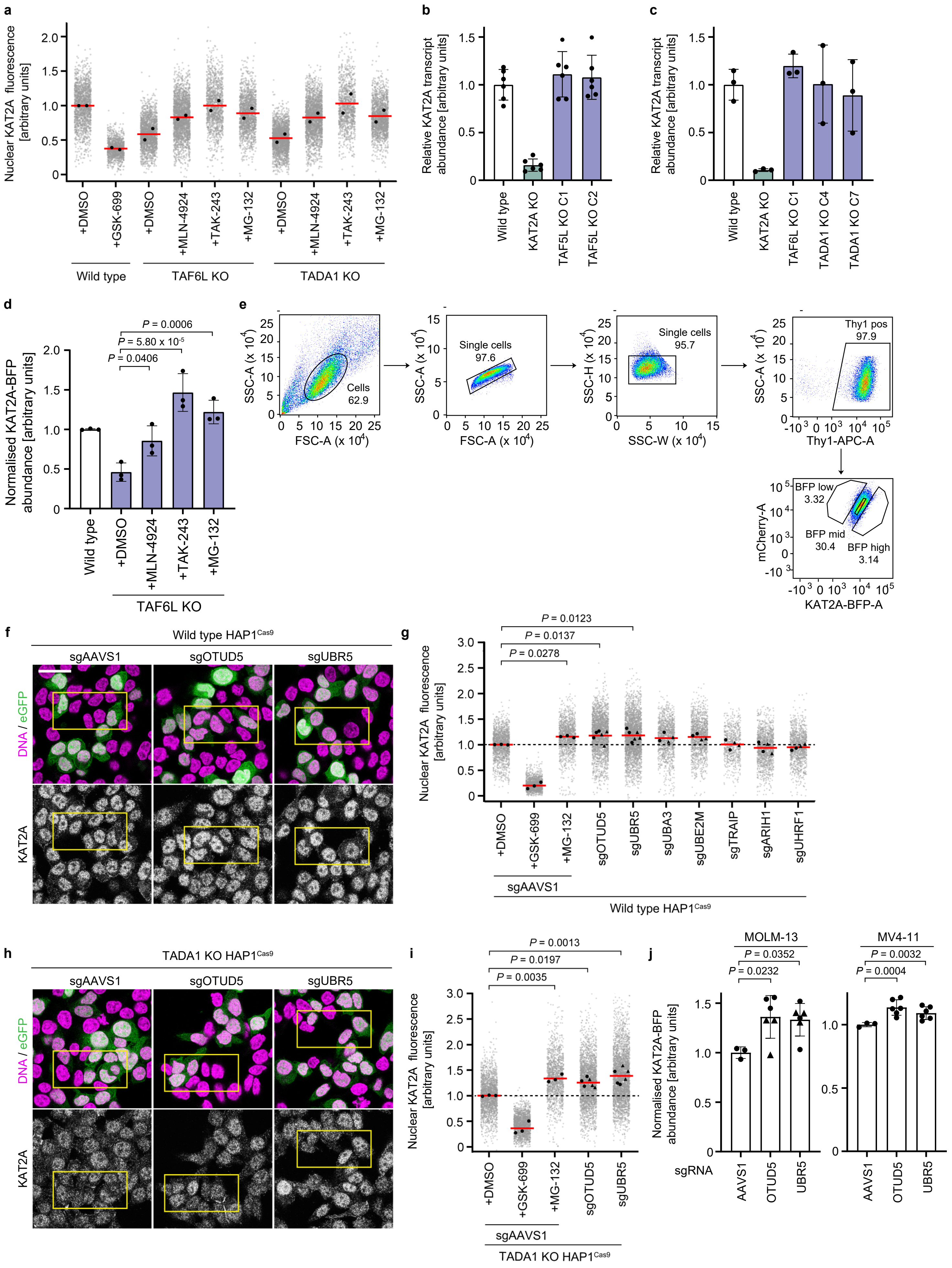

Supplementary Figure 9 | Loss of KAT2A upon SAGA CORE perturbation is proteasome-mediated

Legend on next page.

Supplementary Figure 9 | Loss of KAT2A upon SAGA CORE perturbation is proteasome-mediated

a) Quantification of nuclear KAT2A fluorescence by immunofluorescence for HAP1 cells treated with indicated compounds. **b, c)** RT-qPCR of KAT2A transcript levels for wild type and KO HAP1 cells. **d)** Quantification of normalised KAT2A-BFP abundance by flow cytometry for wild type (white bars) and TAF6L KO (purple bars) cells treated with indicated compounds. **e)** Gating used in the pooled CRISPR screen in Fig. 5e. Numbers indicate cell percentage inside each gate. **f-i)** Wild type or TADA1 KO HAP1^{Cas9} cells were transduced with the indicated eGFP-expressing sgRNAs and fixed for immunofluorescence (5 days post-transduction). **f, h)** Representative immunofluorescence images of KAT2A and DNA/eGFP for wild type **(f)** and TADA1 KO **(h)** HAP1^{Cas9} cells transduced with the indicated sgRNAs. **g, i)** Quantification of nuclear KAT2A fluorescence by immunofluorescence for wild type (**g)** and TADA1 KO **(i)** HAP1^{Cas9} cells transduced with the indicated sgRNAs. **j)** Quantification of KAT2A-BFP abundance by flow cytometry for MOLM-13^{Cas9} or MV4-11^{Cas9} cells transduced with the indicated sgRNAs. iRFP positive cells were analysed 5 days (MOLM-13) or 7 days (MV4-11) post-transduction. Biological replicates: a (n = 2); b (n = 6); c, e, h, i, j (n = 3); f, g (n = 2 apart from sgAAVS1, sgOTUD5, and sgUBR5 (n = 3)). Technical replicates: b-d (n = 3). a, g, i) Grey dots represent the mean of individual nuclei. a-d) Black dots represent the mean of biological replicates. b-d, j) Error bars represent standard deviation, bars indicate the mean for each condition. g, i, j) Two independent sgRNAs are merged for UPS genes. Black dots or triangles represent the mean for each guide. a, g, i) Red bars represent the overall mean of biological replicates. Significance was tested using a one-way ANOVA with a post-hoc Dunnett’s multiple comparison test. Exact p-values are specified in the figure. All images show single Z-slices. Yellow boxes indicate inset regions showing non-transduced (eGFP negative) and transduced (eGFP positive) cells. To aid visualisation the DNA and eGFP channels are not contrast matched. Scale bars: 20 µm.

a

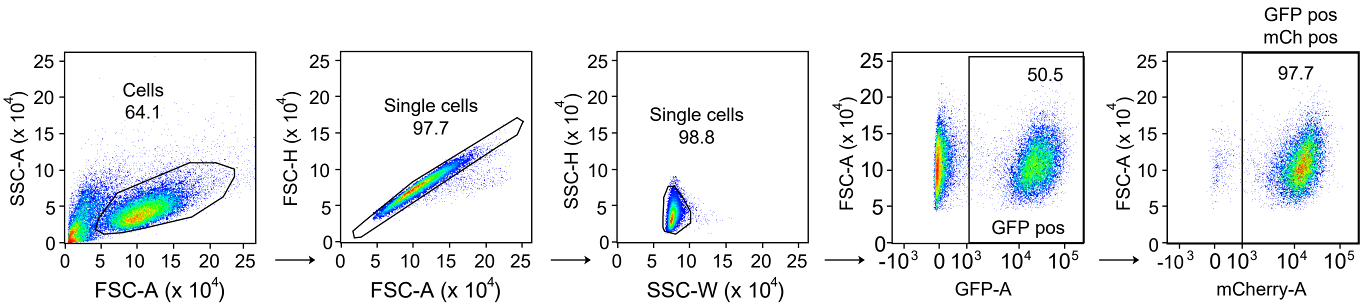

Supplementary Figure 10 | Flow cytometry gating

a) Gating strategy used to analyse data presented in Fig. 1c, Supplementary Fig. 2a-c, Supplementary Fig. 3a, d-f, h, Supplementary Fig. 4b-e, Supplementary Fig. 7c. For all panels apart from Supplementary Fig. 2a-c (where the fraction of GFP positive cells was calculated), the KAT2A-BFP/mCherry ratio of GFP positive cells was measured. Numbers indicate the percentage of cells inside each gate. For stability reporter experiments in Fig. 5c, Fig. 6e, Supplementary Figure 3b, c, Supplementary Figure 9d, the same gating strategy was used, but on GFP negative cells. In Supplementary Fig. 9j cells were gated for iRFP positive rather than GFP positive cells.

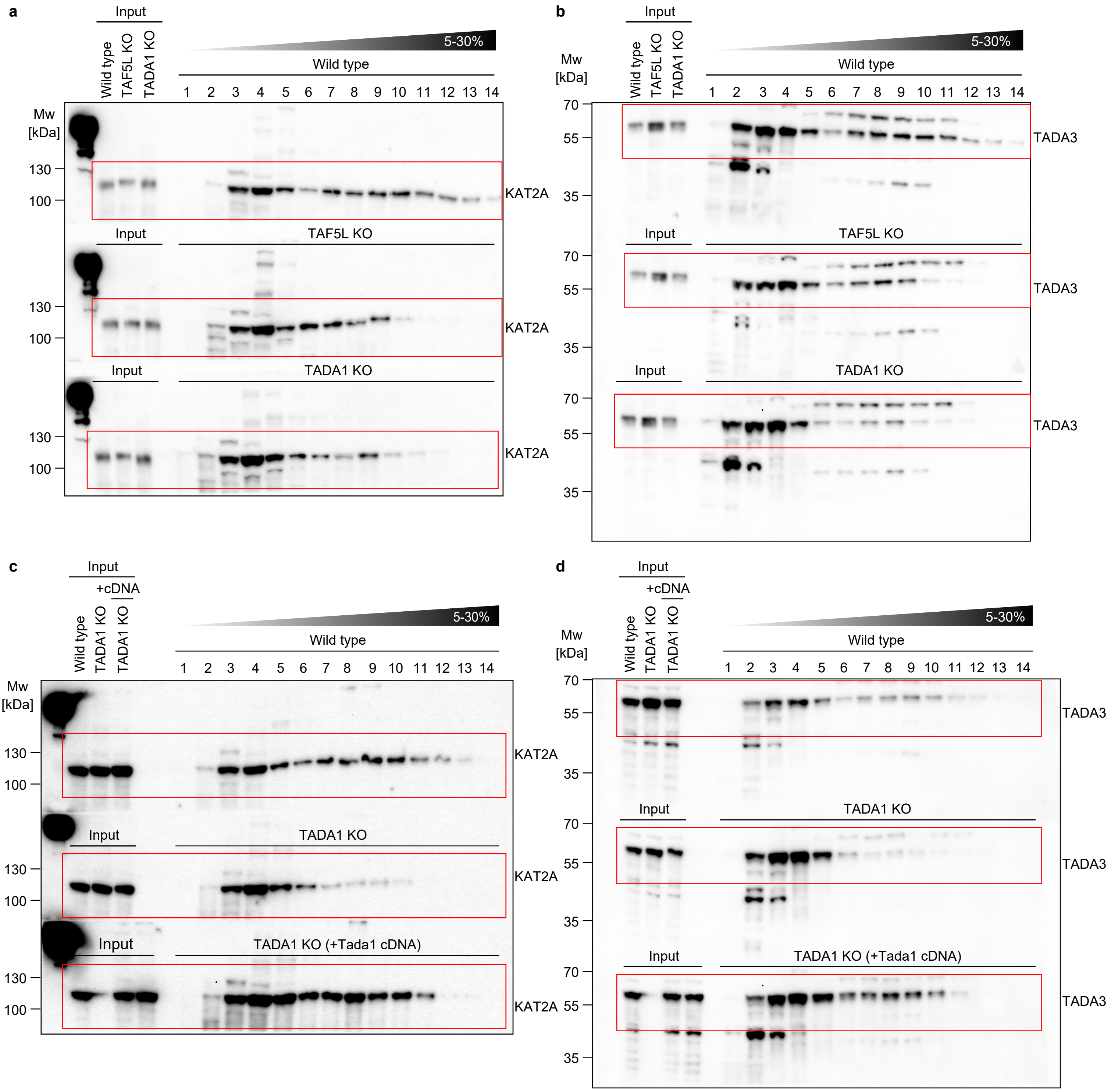

Supplementary Figure 11 | Uncropped western blots

a-d) Uncropped western blots for data shown in Fig. 3b, c, Supplementary Fig. 7a, b. Inputs for each condition were run on the same gel as the target protein and indicate the cell lysates for each condition prior to ultracentrifugation. Membranes were cut at 70 kDa after transfer to allow detection of KAT2A or TADA3. The cropped images used in the requisite figure panels are indicated with red boxes. Numbers indicate each fraction, moving from lowest to highest density sucrose fractions (5 – 30 %). Membranes were developed simultaneously to allow cross-comparison between genotypes. **a)** Uncropped western blot of KAT2A for conditions as shown. Cropped data shown in Fig. 3b. **b)** Uncropped western blot of TADA3 for conditions as shown. Cropped data shown in Fig. 3c. **c)** Uncropped western blot of KAT2A for conditions as shown. Cropped data shown in Supplementary Fig. 7a**. d)** Uncropped western blot of TADA3 for conditions as shown. Cropped data shown in Supplementary Fig. 7b.
