# Supplementary material for: Disruption of the SAGA CORE triggers collateral degradation of KAT2A
Source: Nat Commun. 2026 Apr 20;17:3410. doi: 10.1038/s41467-026-71613-7 (PMC13096341; doi:10.1038/s41467-026-71613-7)
Supplement: Supplementary file 2 — Description of Additional Supplementary Files [file 41467_2026_71613_MOESM2_ESM.pdf]

Description of additional supplementary files

Supplementary Data 1 | sgRNA-expressing plasmids used in this study

Description: List of sgRNA expressing plasmids and their sequences. These plasmids were used in the arrayed SAGA knockout screen, the arrayed ATAC knockout screen, and experiments to validate effectors of KAT2A destabilisation.

Supplementary Data 2 | Reagents used in this study

Description: List of antibodies, cell lines, chemicals, recombinant DNA, critical commercial assays, and software used in this study.

Supplementary Data 3 | Oligonucleotides used in this study

Description: List of oligonucleotides used in this study for genotyping and Sanger sequencing of knockout cells.

Supplementary Data 4 | Raw counts from pooled CRISPR screen to find effectors of KAT2A stability in TAF5L KO cells

Description: Raw counts for each of the 6 sgRNAs used for each gene in the pooled CRISPR screen presented in Fig. 5e.

Supplementary Data 5 | Primers used for RT-qPCR in this study

Description: List of primers used for RT-qPCR of human KAT2A, KAT2B, GAPDH, used in this study.

Supplementary Data 6 | Number of cells analysed by microscopy in this study

Description: Number of cells analysed for each microscopy dataset in this study. Number of cells per replicate, per condition, and in total per experiment are shown.
