# Supplementary material for: Disruption of the SAGA CORE triggers collateral degradation of KAT2A
Source: Nat Commun. 2026 Apr 20;17:3410. doi: 10.1038/s41467-026-71613-7 (PMC13096341; doi:10.1038/s41467-026-71613-7)
Supplement: Supplementary file 4 — Reporting Summary [file 41467_2026_71613_MOESM4_ESM.pdf]

nature portfolio

Corresponding author(s): Davide Seruggia

Last updated by author(s): Mar 15, 2026

Reporting Summary

Nature Portfolio wishes to improve the reproducibility of the work that we publish. This form provides structure for consistency and transparency in reporting. For further information on Nature Portfolio policies, see our Editorial Policies and the Editorial Policy Checklist.

Statistics

For all statistical analyses, confirm that the following items are present in the figure legend, table legend, main text, or Methods section.

n/a	Confirmed	
☐	☒	The exact sample size (*n*) for each experimental group/condition, given as a discrete number and unit of measurement
☐	☒	A statement on whether measurements were taken from distinct samples or whether the same sample was measured repeatedly
☐	☒	The statistical test(s) used AND whether they are one- or two-sided<br>*Only common tests should be described solely by name; describe more complex techniques in the Methods section.*
☒	☐	A description of all covariates tested
☐	☒	A description of any assumptions or corrections, such as tests of normality and adjustment for multiple comparisons
☐	☒	A full description of the statistical parameters including central tendency (e.g. means) or other basic estimates (e.g. regression coefficient) AND variation (e.g. standard deviation) or associated estimates of uncertainty (e.g. confidence intervals)
☐	☒	For null hypothesis testing, the test statistic (e.g. *F*, *t*, *r*) with confidence intervals, effect sizes, degrees of freedom and *P* value noted<br>*Give P values as exact values whenever suitable.*
☒	☐	For Bayesian analysis, information on the choice of priors and Markov chain Monte Carlo settings
☒	☐	For hierarchical and complex designs, identification of the appropriate level for tests and full reporting of outcomes
☒	☐	Estimates of effect sizes (e.g. Cohen's *d*, Pearson's *r*), indicating how they were calculated

Our web collection on statistics for biologists contains articles on many of the points above.

Software and code

Policy information about availability of computer code

Data collection

- Flow cytometry: Flow cytometry data was acquired using n LSR Fortessa instrument (BD Biosciences) with an HTS Loader and analysed using FlowJo v10.10.
- Microscopy: All confocal microscopy experiments, with the exception of Fig 1.D, E, were imaged on a custom Zeiss LSM 980 microscope fitted with an additional Airyscan2 detector, using a 63× NA 1.4 oil DIC Plan-Aphrochromat (Zeiss) objective and ZEN 3.3 Blue 2020 software. The data in experiment 1D, E, was acquired on an Opera Phenix high-content confocal microscope (PerkinElmer) using a 20x air objective and imaging 18 fields of view per well.
- Sequencing data: Novaseq 6000 system (PE 150 mode).
- Western blotting: BioRad imager operated by ImageLab
- qPCR: Bio-Rad CFX Maestro system.

Data analysis

Microscopy
Fields of cells were analysed using a custom automated analysis pipeline written in Python. Before measuring mean nuclear fluorescence of markers of interest, the following criterion was pre-established: only interphase cells were considered for the analysis. For most immunofluorescence experiments, H3S10P was also stained as a mitotic marker, with H3S10P positive cells excluded from the analysis. Size and maximum mean DNA fluorescence filters were applied to the data to also help exclude mitotic cells. A Gaussian blur was applied to the Hoechst channel using the skimage 'filters' module and the Hoechst channel was then segmented using the 'nuclei' module of cellpose (Stringer et al, 2021). Cells which touched the border of the image were excluded using the 'clear_border' functionality from the skimage 'segmentation' module. Individual masks were labelled and applied to each cell in the field. The area of the nuclear mask and mean fluorescence within the nuclear mask was then calculated for each segmented cell, and the data output as a csv file for each input condition. Biological replicates were normalised independently, relative to their own internal positive and negative controls and the normalised data

then merged for the final figure. To account for differences in cell ploidy and the local compaction state of the chromatin, in Fig. 2D the H3K9ac signal level for each condition was determined by dividing the mean nuclear H3K9ac fluorescence of each segmented nucleus by the mean fluorescence of the DNA (stained with Hoechst 33342), to calculate a H3K9ac/Hoechst ratio.

Pooled CRISPR screen
The screen data was processed using the crispr-process-nf Nextflow pipeline (https://github.com/ZuberLab/crispr-process-nf/). In brief, raw FASTQ files were trimmed with cutadapt (v4.4) to remove random barcodes and spacer sequences, followed by demultiplexing based on sample barcodes. Reads were aligned to the custom UPS sgRNA reference library using Bowtie2 (v2.4.5), and sgRNA abundance was quantified with featureCounts (v2.0.1). The resultant count tables (Table S2) were analysed using the crispr-mageck-nf workflow (https://github.com/ZuberLab/crispr-mageck-nf/) for downstream statistical evaluation. Gene-level enrichment was assessed using MAGeCK (v0.5.9) (Li et al., 2014), comparing KAT2Ahigh or KAT2Alow populations to the KAT2Amid reference group, based on median-normalised read counts and replicate-level variance estimation.

Cut&Run
Sequencing reads (150bp paired-end, NovaSeq 6000) were trimmed using TrimGalore and aligned to the human genome, hg38, using Bowtie2 (Langmead & Salzberg, 2012) (--local, --very-sensitive, --no-mixed, --no-discordant, --dovetail). SAMtools (Li et al, 2009) was used to fix mates of paired end reads, merge, sort and index bam files, while a CUT&RUN-specific blacklist (Nordin et al, 2023) was removed from the bam files using BEDtools (Quinlan & Hall, 2010). DeepTools (Ramírez et al, 2016) was then used to build the genome coverage, to compute matrices and plot the profiles of KAT2A aligned around transcriptional start sites.

TMT-expression proteomics
For all DDA measurements, MaxQuant (version 2.6.7.0) with its built-in search engine Andromeda (Cox et al, 2011; Tyanova et al, 2016a) was used for peptide identification and quantification. MS2 spectra were searched against all Swiss-Prot canonical protein sequences obtained from UniProt (UP000005640, downloaded: 19 March 2025), supplemented with common contaminants (built-in option in MaxQuant). Trypsin/P was specified as the proteolytic enzyme. Precursor tolerance was set to 4.5 ppm, and fragment ion tolerance to 20 ppm. The minimal peptide length was defined as seven amino acids, and the “match-between-run” function was disabled. Quantification was performed on the MS3 level. "18plex" (TMTpro) was selected as isobaric labels and the reporter mass tolerance was set to 0.003 Da. For proteome analyses, carbamidomethylated cysteine was set as a fixed modification and oxidation of methionine and N-terminal protein acetylation as variable modifications. The FDR was set to 100%. This search results were then used as input files for Oktoberfest (v.0.8.3) (Picciani et al, 2024). We performed Prosit rescoring (Gessulat et al, 2019) and quantification via the picked-group-FDR approach (v.0.8.1) (The et al, 2022). The Prosit models Prosit_2020_irt_TMT for retention time prediction and Prosit_2020_intensity_TMT for intensity prediction were employed. The output files were filtered at 1% FDR. Perseus was used for data analysis (Tyanova et al, 2016b). Briefly, "common contaminants", "reversed", and "only identified by site" were filtered out, the intensities for each TMT channel were log2-transformed, and median-centric normalised. Samples were then categorically annotated to group replicates together and the "Hawaii plot" function with default values was performed. The obtained matrix was exported for further analyses.

For manuscripts utilizing custom algorithms or software that are central to the research but not yet described in published literature, software must be made available to editors and reviewers. We strongly encourage code deposition in a community repository (e.g. GitHub). See the Nature Portfolio guidelines for submitting code & software for further information.

Data

Policy information about availability of data

All manuscripts must include a data availability statement. This statement should provide the following information, where applicable:

- Accession codes, unique identifiers, or web links for publicly available datasets
- A description of any restrictions on data availability
- For clinical datasets or third party data, please ensure that the statement adheres to our policy

CUT&RUN datasets generated in this study can be accessed on the Gene Expression Omnibus (GEO) database under the series accession number GSE300600. Mass spectrometric raw data files as well as the data analysis output files have been deposited to the ProteomeXchange Consortium via the PRIDE partner repository with the dataset identifier PXD065443. Raw microscopy data are available and will be provided by the corresponding authors on request due to the large file sizes and large number of files involved. IPython notebooks used to analyse data in this manuscript can be found at: https://github.com/seruggialab/Batty_et_al_Nature_Comms_2026.

Research involving human participants, their data, or biological material

Policy information about studies with human participants or human data. See also policy information about sex, gender (identity/presentation), and sexual orientation and race, ethnicity and racism.

Reporting on sex and gender	N/A
Reporting on race, ethnicity, or other socially relevant groupings	N/A
Population characteristics	N/A
Recruitment	N/A
Ethics oversight	N/A

Note that full information on the approval of the study protocol must also be provided in the manuscript.

Field-specific reporting

Please select the one below that is the best fit for your research. If you are not sure, read the appropriate sections before making your selection.

☒ Life sciences ☐ Behavioural & social sciences ☐ Ecological, evolutionary & environmental sciences

For a reference copy of the document with all sections, see nature.com/documents/nr-reporting-summary-flat.pdf

Life sciences study design

All studies must disclose on these points even when the disclosure is negative.

Sample size	No sample-size calculations were performed. Sample sizes were chosen as large as possible while taking into account the experimental effort required to generate the respective data. For microscopy experiments, a minimum of 2 biological replicates were performed as each replicate consisted of multiple wells with hundreds of cells, allowing accurate estimation of the mean and a measure of error. Adequate statistics have been applied throughout the manuscript in order to make sure that the observed effects are significant given the reported sample size.
Data exclusions	For analysis of most microscopy experiments the following pre-criterion was established: only interphase cells were considered for the final analysis. Mitotic cells were excluded based on measurement of H3S10P levels on chromatin.
Replication	Reported experiments were repeated at least twice with consistent results. For all experiments biological replicates have been combined.
Randomization	Randomization was ensured as cells were plated in different plate formats (6, 24 and 96 well plates) and treated in different wells. Moreover, as analysis of data acquired in this paper was largely performed using automated scripts, a blinding of the researcher with respect to individual samples was not necessary as the analysis was applied automatically and similarly to all samples.
Blinding	The researchers were not blinded to the identity of processed samples. However many datasets were generated via automated bulk measurements (e.g. flow cytometry using HTS plate reader, TMT-expression proteomics, DNA sequencing), the introduction of human bias via sample selection can be excluded. Moreover, as analysis of data acquired in this paper was largely performed using automated scripts, a blinding of the researcher with respect to individual samples was not necessary as the analysis was applied consistently to all samples.

Reporting for specific materials, systems and methods

We require information from authors about some types of materials, experimental systems and methods used in many studies. Here, indicate whether each material, system or method listed is relevant to your study. If you are not sure if a list item applies to your research, read the appropriate section before selecting a response.

Materials & experimental systems

n/a	Involved in the study
☐	☒ Antibodies
☐	☒ Eukaryotic cell lines
☒	☐ Palaeontology and archaeology
☒	☐ Animals and other organisms
☒	☐ Clinical data
☒	☐ Dual use research of concern
☒	☐ Plants

Methods

n/a	Involved in the study
☒	☐ ChIP-seq
☐	☒ Flow cytometry
☒	☐ MRI-based neuroimaging

Antibodies

Antibodies used	KAT2A rabbit monoclonal (1:500, IF) Cell Signaling C26A19<br>H3K9ac polyclonal rabbit (1:500, IF) Active Motif 39017<br>HA monoclonal mouse (1:400, IF) Cell Signalling 2367S<br>H3S10P monoclonal mouse (1:5000, IF) Millipore 05806<br>Donkey anti-rabbit Alexa Fluor 488 secondary antibody (1:1000, IF) Molecular Probes A21206<br>Goat anti-rabbit Alexa Fluor 568 secondary antibody (1:1000, IF) Molecular Probes A11011<br>Goat anti-rabbit Alexa Fluor 647 secondary antibody (1:1000, IF) Molecular Probes A21244<br>Anti-KAT2A / GCN5 antibody [EPR21146] - ChIP Grade (1:1000, WB) (1:50, CUT&RUN) abcam ab217876<br>Anti-TADA3 antibody produced in rabbit (1:1000, WB) Sigma HPA042250-100ul<br>anti-KAT2B / PCAF antibody produced in rabbit (1:500, IF) Cell Signaling 3378S<br>Anti-rabbit IgG, HRP-linked antibody (1:10000, WB) Cell Signalling 7074S<br>APC anti-rat CD90/mouse CD90.1 (Thy1.1) Antibody (1:400, FACS) Biolegend 202526
Validation	Antibodies were validated by the provider. KAT2A and TADA3 antibodies were further validated by immunofluorescence or Western blotting using respective KO cells or cells transduced with gRNAs against the respective gene. For KAT2A, cells were also transduced with the KAT2A/B PROTAC GSK-699 and KAT2A levels measured by immunofluorescence. The H3S10P antibody was validated by

measuring fluorescence of mitotic cells compared to interphase cells. The HA antibody was validated by immunofluorescence following treatment of TADA1-HA-dTAG cells with dTAG-v1 and measuring fluorescence over time.

Eukaryotic cell lines

Policy information about cell lines and Sex and Gender in Research

Cell line source(s)	HAP-1 cells were obtained from Horizon Discovery; HEK293T were obtained from Takara; NALM6, MOLM13 and MV411 were obtained from ATCC
Authentication	Cell lines were authenticated by the provider
Mycoplasma contamination	Cells were tested weekly for mycoplasma contamination using MycoAlert® Mycoplasma Detection Kit (Lonza)
Commonly misidentified lines (See ICLAC register)	N/A

Plants

Seed stocks	N/A
Novel plant genotypes	N/A
Authentication	N/A

Flow Cytometry

Plots

Confirm that:

- [x] The axis labels state the marker and fluorochrome used (e.g. CD4-FITC).
- [x] The axis scales are clearly visible. Include numbers along axes only for bottom left plot of group (a 'group' is an analysis of identical markers).
- [x] All plots are contour plots with outliers or pseudocolor plots.
- [x] A numerical value for number of cells or percentage (with statistics) is provided.

Methodology

Sample preparation	For flow cytometry, the medium was removed from each well, cells washed twice with 100 µl PBS, and incubated with 100 µl Accutase for 5 – 10 min at room temperature. Cells were resuspended in the plate and 50 µl of the cell suspension transferred to a V-bottomed 96-well plate (Thermo Fisher, 277143) containing 50 µl of FACS buffer (PBS containing 2 % FCS, 2 mM EDTA pH 8.0 (Thermo Fisher, AM9260G)).
Instrument	BFP, GFP and mCherry fluorescence was measured for each well using an LSR Fortessa instrument (BD Biosciences) with attached HTS Loader.
Software	Flow cytometry data was analysed using FlowJo V10.10.0.
Cell population abundance	N/A

Gating strategy

Representative plots of the gating strategy used for FACS in the pooled CRISPR screen presented are shown in Fig. S4E. Numbers indicate the percentage of cells inside the respective gate. TAF5L KO HAP1Cas9 cells expressing the KAT2A stability reporter were gated using the following logic: 1) FSC-A vs SSC-A to gate cells, 2) FSC-A vs FSC-H and SSC-H vs SSC-W to gate single cells, FSC-A vs Thy1-APC-A to identify sgRNA expressing cells, and then KAT2A-BFP-A vs mCherry-A to sort KAT2A-BFPlow, KAT2A-BFPmid, and KAT2A-BFPhigh populations. The gating for KAT2A-BFP was adjusted dynamically to sort the top and bottom 3 % of KAT2A-BFP expressing cells. In Fig. 1c, wild type HAP1Cas9 cells expressing the KAT2A stability reporter were gated in the following way: 1) FSC-A vs SSC-A to gate cells, 2) FSC-A vs FSC-H and SSC-H vs SSC-W to gate single cells, FSC-A vs GFP-A to gate eGFP positive cells. The KAT2A-BFP/mCherry ratio was measured for eGFP positive cells for each guide. An example of the gating in shown in Supplementary Fig. 1a. This gating was used in Fig. 1c, Extended Data Fig. 2a-c, Extended Data Fig. 3a, d-f, h, Extended Data Fig. 4b-e, Extended Data Fig. 7c. For stability reporter experiments in Fig. 5c, Fig. 6e, Extended Data Fig. 9d, the same gating strategy was used, with the exception of gating for eGFP positive cells as the experiment was performed on eGFP negative cells. In Extended Data Fig. 9j cells were gated for iRFP positive rather than eGFP positive cells.

[x] Tick this box to confirm that a figure exemplifying the gating strategy is provided in the Supplementary Information.
